# Hidden lymphoma in the appendix: a case of primary appendiceal diffuse large B-cell lymphoma presenting as acute appendicitis

**DOI:** 10.1093/jscr/rjad289

**Published:** 2023-06-01

**Authors:** Paola Solis-Pazmino, Luana Vasques, Pedro Maldonado, João Dias Lunardi, Mateus Schneider, Andre Gorgen

**Affiliations:** Surgery Department, Santa Casa de Misericórdia in Porto Alegre (SCMPA), Porto Alegre, Brazil; Surgery Department, Santa Casa de Misericórdia in Porto Alegre (SCMPA), Porto Alegre, Brazil; Surgery Department, Santa Casa de Misericórdia in Porto Alegre (SCMPA), Porto Alegre, Brazil; Surgery Department, Santa Casa de Misericórdia in Porto Alegre (SCMPA), Porto Alegre, Brazil; Surgery Department, Santa Casa de Misericórdia in Porto Alegre (SCMPA), Porto Alegre, Brazil; Surgery Department, Santa Casa de Misericórdia in Porto Alegre (SCMPA), Porto Alegre, Brazil

## Abstract

The occurrence of diffuse large B-cell lymphoma (DLBCL) of the appendix presenting as acute appendicitis is rare, constituting only a minuscule portion (0.015%) of gastrointestinal lymphoma cases. We present a case of a 55-year-old woman that was admitted to the emergency department with symptoms of acute appendicitis and underwent an interval laparoscopic appendectomy with a right hemicolectomy and lymph node resection. Pathological examination revealed the presence of DLBCL in the appendix. The patient is undergoing chemotherapy.

## INTRODUCTION

Diffuse large B-cell lymphoma (DLBCL) of the appendix presenting as acute appendicitis is rare, representing only 0.015% of all gastrointestinal lymphoma cases [1]. This cancer explicitly affects the appendix, a small pouch-like organ located at the beginning of the large intestine. It is a subtype of non-Hodgkin lymphoma, a group of cancers originating from lymphocytes. In the case of DLBCL, the B-cells grow out of control and form a tumor in the appendix [2].

This type of cancer can metastasize to other body parts if left untreated, following an aggressive path of dissemination and organ invasion [3]. The standard course of treatment for DLBCL of the appendix usually involves a combination of different therapies, such as surgery, chemotherapy, radiation therapy and immunotherapy [4]. The treatment plan is tailored to the individual patient based on the disease presentation and their unique circumstances.

Early detection and prompt treatment can significantly improve the prognosis for patients with appendiceal DLBCL [3]. Regular check-ups and immediate medical attention to any unusual symptoms are crucial to diagnosing this type of cancer in its early stages and improving the chances of a successful outcome. Generally, patients diagnosed early and receiving appropriate treatment have a better prognosis than those diagnosed later.

## CASE REPORT

A 55-year-old woman with a previous surgical history of cholecystectomy and myomectomy was admitted to the digestive surgery service after experiencing 2 months of pain in the right lower quadrant. She denied fever, weight loss or change in defecation.

On physical examination, the patient reported mild tenderness in the right iliac fossa upon palpation but denied irradiation of the pain or other sign of peritonism. The cardiorespiratory examination showed no abnormal findings.

**Figure 1:**
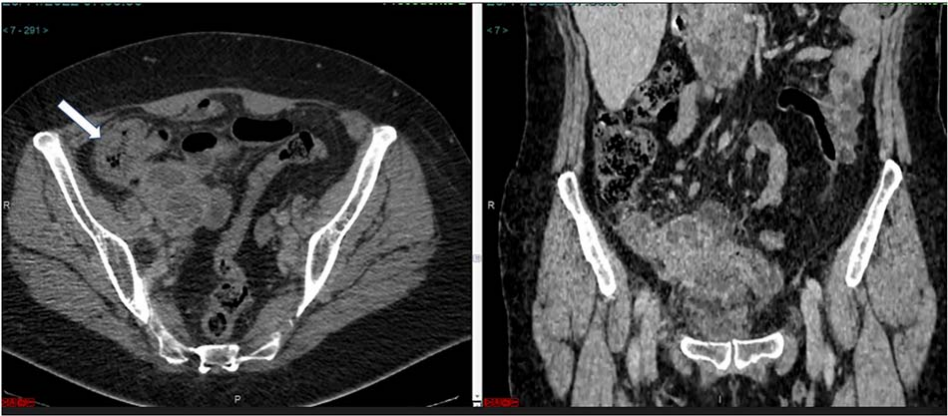
(**a**) Axial CT section of the abdomen showing signs of mucocele of the appendix with intraluminal air. (**b**) The coronal CT section of the appendix shows that the anterior wall of the appendix had an irregular thickening close to the transition of the middle/distal third.

The abdominal computed tomograCT scan showed an enlargement of the cecal appendix with a maximum diameter of 2.8 cm. Irregularities were identified in the lower contour of the appendix, with liquid accumulation and thickened wall measuring 1.6 × 1.4 × 1.1 cm (Fig. 1). Laboratory testing revealed an elevated C-reactive protein level of 216 mg/L and high white blood cells count. Renal function and electrolytes were within the normal range.

### Treatment

The patient underwent a laparoscopic exploration. During the operation, at the level of the right iliac fossa, a sizeable appendicular mass measuring 9.2 cm × 3.4 cm × 3.0 cm was noted with a contiguous invasion of the right colon and two segments of the small bowel. Because of multiple adhesions at the right iliac fossa, the procedure was converted into a laparotomy through a midline incision.

The two loops of the small bowel involved in the lesion had >90% of their circumference compromised, resulting in intestinal obstruction. Surgery proceeded with a right hemicolectomy sent for transoperative freezing examination, demonstrating atypical lymphocytosis. Pelvic lymphadenectomy of the internal iliac chain was performed, and the material was sent for histopathologic analysis with a pre-diagnosis of lymphoproliferative neoplasia.

**Figure 2:**
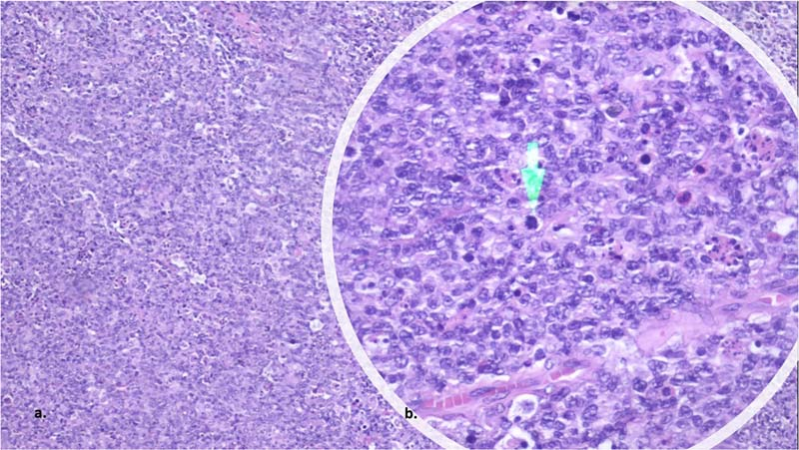
The histopathology showed a lymphoproliferative lesion of large and atypical cells with necrosis and hemorrhage, suggesting high-grade non-Hodgkin’s lymphoma.

**Figure 3:**
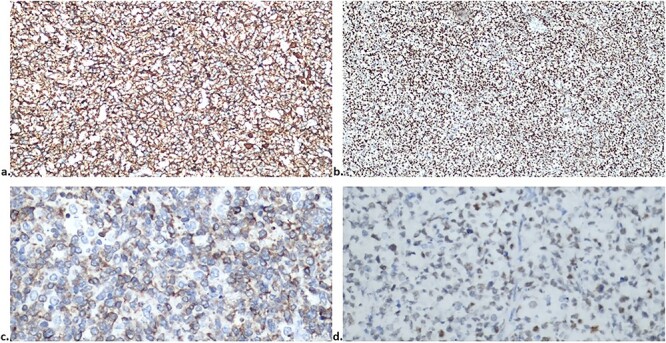
Immunohistochemistry of the lesion showed CD20, MUM1, BCL2 and BCL6 positive markers (**a**, **b**, **c** and **d**, respectively).

The anatomopathological examination of the colon demonstrated a lymphoproliferative lesion of large and atypical cells with necrosis and hemorrhage, suggesting a high-grade non-Hodgkin’s lymphoma (Fig. 2a and b). Immunohistochemistry of the lesion resulted in CD20, MUM1, BCL2 and BCL6 positive markers (Fig. 3a–d), compatible with DLBCL of the non-germinative center type. In the postoperative period, the patient had no complications and was discharged in 1 day. Then, the patient began her first six chemotherapy courses with cyclophosphamide, doxorubicin HCl, vincristine and rituximab (R-CHOP) every 21 days.

### Differential diagnosis

Mucocele of the appendix is a condition with symptoms that can mimic other illnesses. Thus, it is crucial to perform a differential diagnosis to eliminate other possible causes of the patient’s symptoms. Possible differential diagnoses for mucocele of the appendix include appendicitis, ovarian cyst, diverticulitis, Crohn’s disease and gastrointestinal tumors. A comprehensive evaluation that incorporates the patient’s medical history, physical examination, imaging studies and laboratory tests may be required to make an accurate diagnosis. Performing a precise diagnosis is essential to achieve optimal treatment outcomes and minimize the likelihood of complications.

## DISCUSSION

Primary appendiceal lymphoma (PAL) is a rare form of gastrointestinal lymphoma, accounting for just 0.015% of all diagnoses [5]. The present case study describes a DLBCL initially misdiagnosed as acute appendicitis and treated with a laparoscopic appendectomy. After completing the R-CHOP-21 chemotherapy regimen, the patient showed complete remission, as confirmed by a follow-up PET scan [1].

Symptoms of appendiceal lymphoma may include abdominal pain, bloating, constipation or diarrhea, but some patients may be asymptomatic, and the condition may be discovered incidentally. Depending on the stage and type of cancer, patient health and personal preferences, treatment options may include surgery, chemotherapy, radiation therapy or a combination of treatments [6].

Available treatment options are limited. However, recent studies have shown that combining surgical resection and postoperative chemotherapy can offer promising outcomes for patients with localized intestinal DLBCL [7]. The surgical resection involves the removal of the affected appendix, followed by the administration of CHOP chemotherapy, which is highly effective in eradicating cancer cells. Surgical resection and CHOP chemotherapy have demonstrated a low recurrence rate, with no evidence of cancer growth reported in patients 6 months after the operation [8]. This is a positive sign, indicating that this treatment approach can lead to long-term remission in some cases.

The prognosis for PAL varies based on the type and stage of the disease and the patient’s health. Patients with early-stage disease tend to have a better prognosis than those with advanced disease. A retrospective analysis of 116 patients with PAL by Ayub *et al*. [4] revealed an average age at diagnosis of 48 years, average overall survival of 185 months and a 5-year survival rate of 67%. There was no significant difference in overall survival based on gender, race or histologic subtype. Still, increasing age at diagnosis was significantly associated with an increased risk of death in multivariate analysis. Meanwhile, factors such as gender, race, tumor histology, disease stage and type of resection were not significantly linked to overall survival.

Despite these promising results, it is essential to note that each patient’s case is unique. Close monitoring and follow-up are essential to address potential complications or recurrences [9] promptly. With clear guidelines for treating primary appendiceal lymphoma, healthcare providers can work closely with their patients to develop a personalized and effective treatment plan.

## CONCLUSION

Lymphoma of the appendix is a rare type of cancer that requires prompt diagnosis and treatment. Patients should seek medical attention if they experience symptoms and undergo regular medical check-ups to monitor their health and catch any potential issues early.

## CONFLICT OF INTEREST STATEMENT

None declared.

## INFORMED CONSENT

Informed consent was obtained from the patient to be included in the study.
